# Adult attention-deficit/hyperactivity disorder symptoms, cognitive dysfunction and quality of life in high-dose use of benzodiazepine and Z-drug

**DOI:** 10.1007/s00702-020-02285-w

**Published:** 2020-12-17

**Authors:** Angela Federico, Elisa Mantovani, Rebecca Casari, Anna Bertoldi, Fabio Lugoboni, Stefano Tamburin

**Affiliations:** 1 Department of Neurosciences, Biomedicine and Movement Sciences, University of Verona, Piazzale Scuro 10, 37134 Verona, Italy; 2 Addiction Medicine Unit, Department of Medicine, Verona University Hospital, Piazzale Scuro 10, Verona, Italy

**Keywords:** Attention-deficit/hyperactivity disorder (ADHD), Benzodiazepine (BZD), Cognition, Patient-centered outcomes, Quality of life (QoL), Substance-use disorder (SUD)

## Abstract

High-dose use of benzodiazepines (BZDs) and Z-drugs was found to be associated with adult attention deficit/hyperactivity disorder (ADHD) and multidomain cognitive deficits, but the interplay between these factors and its effect on quality of life (QoL) is unclear. We explored (a) whether cognitive dysfunction differs in high-dose BZD/Z-drug users with and without adult ADHD and (b) the impact of cognitive deficits and adult ADHD on QoL in this substance-use disorder (SUD). From January 2015 to December 2019, we recruited 207 high-dose BZD/Z-drug users seeking treatment. We assessed the presence of adult ADHD with a screening tool, which was validated in SUD patients, and collected demographic, clinical and QoL data from the 76 included patients. A neuropsychological battery explored five cognitive domains. We found that: (a) screening for adult ADHD was frequently positive; (b) Short Form-36 (SF-36), a self-administered QoL questionnaire, was worse than the general population and worse in patients positive (ADHD+) vs. those negative (ADHD−) to ADHD screening tool; (c) executive function was significantly worse in ADHD+ than ADHD− patients; (d) some SF-36 dimensions were negatively influenced by executive dysfunction; (e) multivariate analysis showed an interplay between adult ADHD and cognitive dysfunction in worsening QoL. We documented a complex interplay between adult ADHD, cognitive dysfunction and QoL in high-dose BZD/Z-drug users. Assessing adult ADHD, neuropsychological measures and QoL may offer a full scenario of these patients, who are frequently impaired in everyday activities. Future research should explore whether pharmacological treatment might improve cognitive dysfunction and QoL in this SUD.

## Introduction

Benzodiazepines (BZDs) and related Z-drugs (i.e., zolpidem, zopiclone, eszopiclone, and zaleplon) are positive allosteric modulators of the gamma-amino-butyric acid type A receptor that are prescribed for the treatment of anxiety and insomnia (Soyka 2017). Despite guidelines stipulate that BZDs and Z-drugs should be used for short periods of time, their long-term use is reported in 6–76% and dependence in 3–4% of patients (Kurko et al. 2015; Soyka 2017).

The estimated prevalence of long-term use of BZDs and Z-drugs at high doses ranges from 0.06 to 0.16% of the population in Europe (Ohayon and Lader 2002; Petitjean et al. 2007). We have previously documented that high-dose BZD and Z-drug use (i.e., ≥ 5 times the recommended maximum daily dose; Liebrenz et al. 2015) is associated with worse quality of life (QoL; Tamburin et al. 2017b) and cognitive dysfunction involving multiple domains (Federico et al. 2017).

Attention-deficit/hyperactivity disorder (ADHD) is a neurodevelopmental disorder that has 6–9% prevalence in children (Polanczyk and Rohde, 2007), and may persist in adults with 2.5–5% prevalence (Volkow and Swanson, 2013; Bonvicini et al. 2016). Adult ADHD has been reported to be more common in people with SUD than the general population (van Emmerik-van Oortmerssen et al. 2012; van de Glind et al. 2014), and to be associated with worse SUD course (Mariani and Levin 2007; Liao et al. 2017; Rodríguez-Cintas et al. 2018; Lugoboni et al. 2020a) and reduced QoL in the general population (Ahnemark et al. 2018) and in patients with SUD (Liao et al. 2017).

Routine assessment of ADHD in adult people with SUD is helpful but may be complex because of the long diagnostic interview that should include a retrospective investigation of childhood symptoms (Tamburin et al. 2017c) and different diagnostic criteria across DSM versions (van de Glind et al. 2013). A validated screening tool for adult ADHD with good accuracy and short application time might be used in the clinical setting (van de Glind et al. 2013). We have previously documented that a screening test for adult ADHD may be positive in approximately one-third of high-dose BZD and Z-drug users (Tamburin et al. 2017c), and that adult ADHD is associated with worse QoL in this population (Lugoboni et al. 2020b).

ADHD is associated with substantial deficits across a variety of cognitive domains, including working memory, reaction time variability, response inhibition, intelligence/achievement, planning/organization, and vigilance (Pievsky and McGrath 2018). Multidomain cognitive deficits in adult ADHD have been suggested to be explained by impairment of basic processes (i.e., processing speed and distractibility; Butzbach et al. 2019) and sustained attention (Tucha et al. 2017).

Data on the interaction between adult ADHD, cognition and QoL in high-dose BZD/Z-drug users are lacking. This paper is aimed to answer two questions. The first is whether cognitive dysfunction differs in high-dose BZD and Z-drug users with and without adult ADHD. The second is to explore the impact of cognitive deficits and adult ADHD on QoL in this SUD population. To these aims, we recruited a group of high-dose BZD/Z-drug users seeking treatment, assessed the presence of adult ADHD with a screening tool, which was validated in SUD patients (van de Glind et al. 2013), and collected demographic, clinical, cognitive and QoL data. Since coexisting SUD to other drugs, neurologic or major psychiatric disorders might influence both cognitive function and QoL, these conditions were ruled out.

## Methods

### Patients

We recruited 207 high-dose BZD or Z-drug users (94 men and 113 women), who were admitted to the Department of Medicine, Addiction Medicine Unit, Verona University Hospital, Italy from January 2015 to December 2019 for detoxification with slow subcutaneous infusion of flumazenil (Tamburin et al. 2017a).

**Fig. 1:**
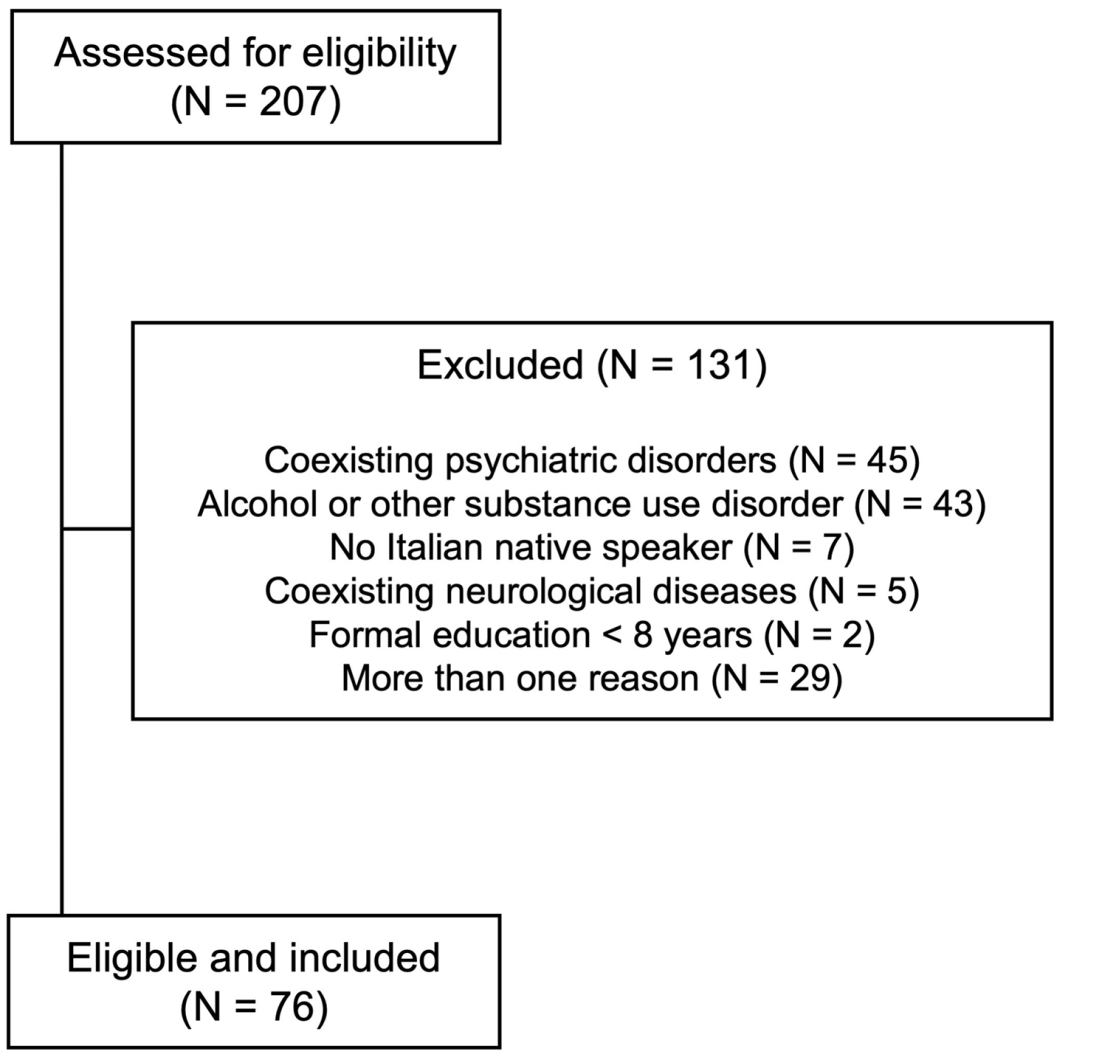
Flow diagram of the study and reasons for patients’ exclusion

The inclusion criteria were: (a) age > 18 years, (b) formal education ≥ 8 years, (c) Italian as mother language, (d) normal or corrected-to-normal vision, (e) no hearing loss, (f) no acute drug intoxication, (g) normal overall cognition documented by a Mini Mental State Examination score > 24/30, (h) no neurological diseases that might interfere with cognition, (i) no major psychiatric disorders, and (j) no concurrent alcohol use or other SUD (Federico et al. 2017). The diagnosis of psychiatric disorders was based on screening tests, diagnostic interviews, and previous psychiatric evaluations, when available. Among major psychiatric disorders, anxiety disorders and mild depression were not considered as exclusion criteria because they are common in patients taking BZDs/Z-drugs and we ruled out only more severe psychiatric conditions (i.e., severe psychoses and personality disorders). After selection, 76 patients (34 men, 42 women; age 43.2 ± 10.0 years, median 43; education 12.1 ± 3.3 years, median 13) were included (Fig. 1).

High-dose BZD/Z-drug dependence was defined according to DSM-IV-TR criteria, with use lasting > 6 months, a daily dosage ≥ 5 times the recommended maximum intake (i.e., ≥ 50 mg of daily diazepam dose equivalent, DDDE), and/or otherwise problematic use of BZDs/Z-drugs, such as mixing molecules, escalating dosage, obtaining them by illegal means and using them to enhance the effect of other substances (Liebrenz et al. 2015; Tamburin et al. 2017a, c).

We collected and analyzed demographic (sex, age, education: years), and clinical variables (BZD/Z-drug active principle, BZD/Z-drug dosage: DDDE, mg; BZD/Z-drug use duration: months; tobacco smoke; depression; anxiety). The dosage of BZD/Z-drug was based on patient’s self-report.

The study was conducted according to the Declaration of Helsinki and approved by the Ethics Committee of the Provinces of Verona and Rovigo based at Verona University Hospital (approval ID: 683CESC). All patients gave written informed consent for participation to the study and for data to be published. All the collected data were anonymized with a coding procedure.

### Adult ADHD screening

Before starting detoxification, patients were screened for adult ADHD with the self-administered World Health Organization Adult six-question ADHD Self-Report Scale version 1.1 (ASRS v1.1) Symptom Checklist Part A (Kessler et al. 2005). The ASRS v1.1 Symptom Checklist Part A with a cutoff ≥ 4 was reported to have good sensitivity and k and very high specificity and total classification accuracy (Kessler et al. 2005; Tamburin et al. 2017c) and to represent a sensitive screener for identifying possible ADHD patients with very few missed cases in patients with SUD (van de Glind et al. 2013).

### Depression and anxiety measures

The Beck Depression Inventory II (BDI-II), a 21-item self-administered questionnaire (score 0–3 for each item, cutoff for moderate to severe depression 28), was used to measure the severity of depressive symptoms during the previous 2 weeks (Federico et al. 2017). The State Trait Anxiety Inventory form Y (STAI-Y), which is composed of two 20-item self-applied questionnaires, was used to measure state and trait anxiety. Each STAI-Y item is scored on a 1–4 Likert-type format, and the cutoff for mild anxiety is 40 (Federico et al. 2017).

### Neuropsychological evaluation

Before detoxification, patients underwent a neuropsychological battery to explore verbal, visuospatial and working memory, attention, and executive function (Federico et al. 2017; Cecchini et al. 2019). For each cognitive domain, we chose a test with available normal distribution of the normative data to calculate *Z*-scores (see “Statistical analysis” section).

Verbal memory was assessed with the Italian versions of the Digit Span Forward Test (DSFT), which measures short-term memory. For DSFT, subjects are asked to repeat progressively longer digit series starting from three up to the longest series they can remember (Monaco et al. 2013).

Visuospatial memory was assessed with the Rey–Osterrieth Complex Figure Test (ROCF), where subjects are asked to copy a complex bidimensional figure (immediate recall) and then redraw it after a 10-min delay (delayed recall; Caffarra et al. 2002).

Working memory was assessed with the Digit Span Backward Test (DSBT), which is the same as DSFT, but subjects are asked to recall the digit series in reverse of the presented order (Monaco et al. 2013).

Attention was assessed with the Trail Making Test Part A (TMT-A), which explores selective attention and visuospatial exploration, by asking the subject to draw lines sequentially connecting 25 encircled numbers (Amodio et al. 2008).

Executive function was evaluated with the Trail Making Test Part B (TMT-B), which is similar to TMT-A, except that the task evaluates mental flexibility and task switching by asking the subjects to alternate between numbers and letters (Amodio et al. 2008).

### QoL measures

At admission, before starting detoxification, patients underwent two self-administered QoL questionnaires, namely the Short Form-36 (SF-36) and the General Health Questionnaire-12 (GHQ-12).

The SF-36 is a generic QoL scale composed of 36 items that are grouped into 8 dimensions: physical functioning (PF), role physical (RP), bodily pain (BP), general health (GH), vitality (VT), social functioning (SF), role emotional (RE), mental health (MH), with scores ranging from 0 (worst score) to 100 (best score) for each dimension (Brazier et al. 1992).

The GHQ-12 explores psychological health and is composed of 12 questions on mood states over the previous 2 weeks (Goldberg and Hillier 1979). GHQ-12 was scored on a two-point scale, resulting in 0–12 total score range with higher values indicating more severe psychological distress (Goldberg and Hillier 1979), and a cutoff value of ≥ 4 (Piccinelli et al. 1993).

### Statistical analysis

All tests were carried with the IBM SPSS version 20.0 statistical package. The normality of variable distribution was analyzed with the Skewness–Kurtosis test. The Pearson’s *χ*^2^ test was used for categorical variables. The unpaired *t* test and the non-parametrical Mann–Whitney *U* test were used for continuous variables. Neuropsychological scores were reported as *Z*-scores according to the formula: *Z*-score = (measured value − mean value according to age and/or education)/standard deviation according to age and/or education. Negative and positive values indicated worse and better performance than the normal population, respectively. *Z*-scores were computed for tests with normal distribution in the normative sample, i.e., DSFT and TMT-A/B time (s), DSBT, ROCF delayed recall (Carlesimo et al. 2002; Mondini et al. 2011; Monaco et al. 2013). The potential confounder effect of covariates (sex, age, education, DDDE, BZD/Z-drug use duration, depression, anxiety) on neuropsychological outcomes was explored with a multivariate generalized linear model (Federico et al. 2017). Correlations between neuropsychological and SF-36 measures were explored with the Spearman’s ρ correlation coefficient. Multivariate backward linear regression model analysis was applied to SF-36 dimensions (continuous dependent variables). Logistic regression model analysis was used for GHQ-12 (binary dependent variable: ≥ 4, < 4), and the results were expressed as odd ratios (ORs) and 95% confidence intervals (CI). *p* < 0.05 (two-tailed) was taken as the significance threshold for all the tests.

## Results

### Demographic and clinical variables

The ASRS v1.1 Symptom Checklist Part A was positive (ADHD +) in 30 (39.5%) and negative (ADHD−) in 46 (60.5%) of the 76 included patients.

Demographic and clinical variables did not differ between ADHD + and ADHD− patients (Table 1).

**Table 1:** Demographic and clinical characteristics of the patients according to the ASRS v1.1 Symptom Checklist Part A

	ADHD+ (*N* = 30)	ADHD− (*N* = 46)	*p* value
Demographic			
Sex (men/women)^a^	15 (50.0%)/15 (50.0%)	19 (41.3%)/27 (58.7%)	0.46
Age^b^	41.9 ± 9.9; 41	44.1 ± 10.1; 43.5	0.23
Education (years)^b^	12.0 ± 3.5; 13	13.1 ± 3.6; 13	0.34
Clinical			
BZD/Z-drug active principle^c^			0.90
Lormetazepam	19 (63.3%)	29 (63.0%)	
Zolpidem	5 (16.7%)	8 (17.4%)	
Alprazolam	3 (10.0%)	5 (10.9%)	
Clonazepam	2 (6.7%)	1 (2.2%)	
Lorazepam	1 (3.3%)	2 (4.3%)	
Triazolam	0 (0%)	1 (2.2%)	
BZD/Z-drug dosage, (DDDE, mg)^b^	473.5 ± 345.1; 375	367.6 ± 357.7; 250	0.23
BZD/Z-drug use duration (months)^b^	125.4 ± 107.6; 78	115.6 ± 92.4; 120	0.88
Tobacco smoke (yes/no)^a^	18 (60.0%)/12 (40.0%)	28 (60.9%)/18 (39.4%)	0.94
Depression (BDI–II)^b^	24.4 ± 11.1; 25	26.5 ± 12.0; 30	0.28
State anxiety (STAI-Y)	40.0 ± 5.3; 40	40.8 ± 6.7; 42	0.48
Trait anxiety (STAI-Y)	45.7 ± 11.0; 44	44.8 ± 8.5; 45	0.87

ADHD+/ADHD−: positive/negative screening for adult ADHD according to the Adult ADHD Self-Report Scale version 1.1 (ASRS v1.1) Symptom Checklist Part A

*ADHD* attention-deficit/hyperactivity disorder,* BDI-II* Beck Depression Inventory II, *BZD* benzodiazepine, *DDDE* daily diazepam dose equivalent, *STAI-Y* State Trait Anxiety Inventory form

^a^*N* (% of row)

^b^Mean ± S.D; median

^c^*N* (% of column)

### Neuropsychological measures

**Fig. 2:**
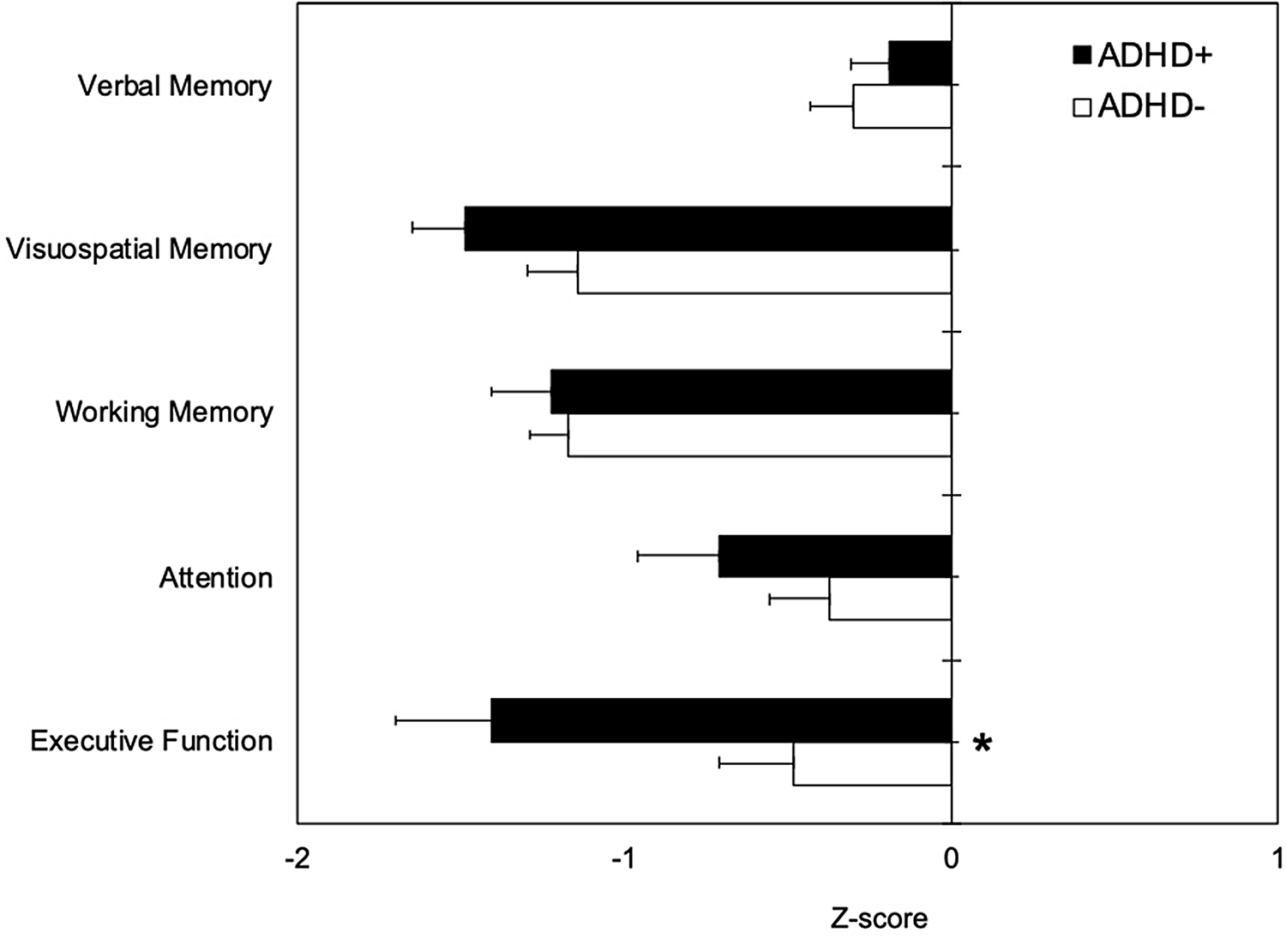
Neuropsychological outcomes (*Z*-scores) in patients positive (ADHD+ , black boxes) and negative (ADHD−, white boxes) to the ASRS v1.1 Symptom Checklist Part A. Negative and positive *Z*-scores indicate worse and better performance than the average value of the normal population, respectively. Horizontal error bars equal 1 S.E.M. * marks *p* < 0.05 for ADHD+ vs. ADHD– comparison

Among neuropsychological measures, only executive function was significantly worse in the ADHD + (− 1.41 ± 1.62, median − 1.28) than the ADHD− group (− 0.48 ± 1.61, median − 0.27; *p* = 0.024), while the other domains (verbal memory: *p* = 0.78; visuospatial memory: *p* = 0.16; working memory: *p* = 0.83; attention: *p* = 0.33) did not significantly differ between the two groups (Fig. 2). Repeated statistical analysis with multivariate generalized linear model including potential covariates confirmed the significant ADHD + vs ADHD− difference for executive function *Z*-score (*p* = 0.028), while the other domains were not significant (verbal memory: *p* = 0.18; visuospatial memory: *p* = 0.70; working memory: *p* = 0.53; attention: *p* = 0.12).

### QoL measures

**Fig. 3:**
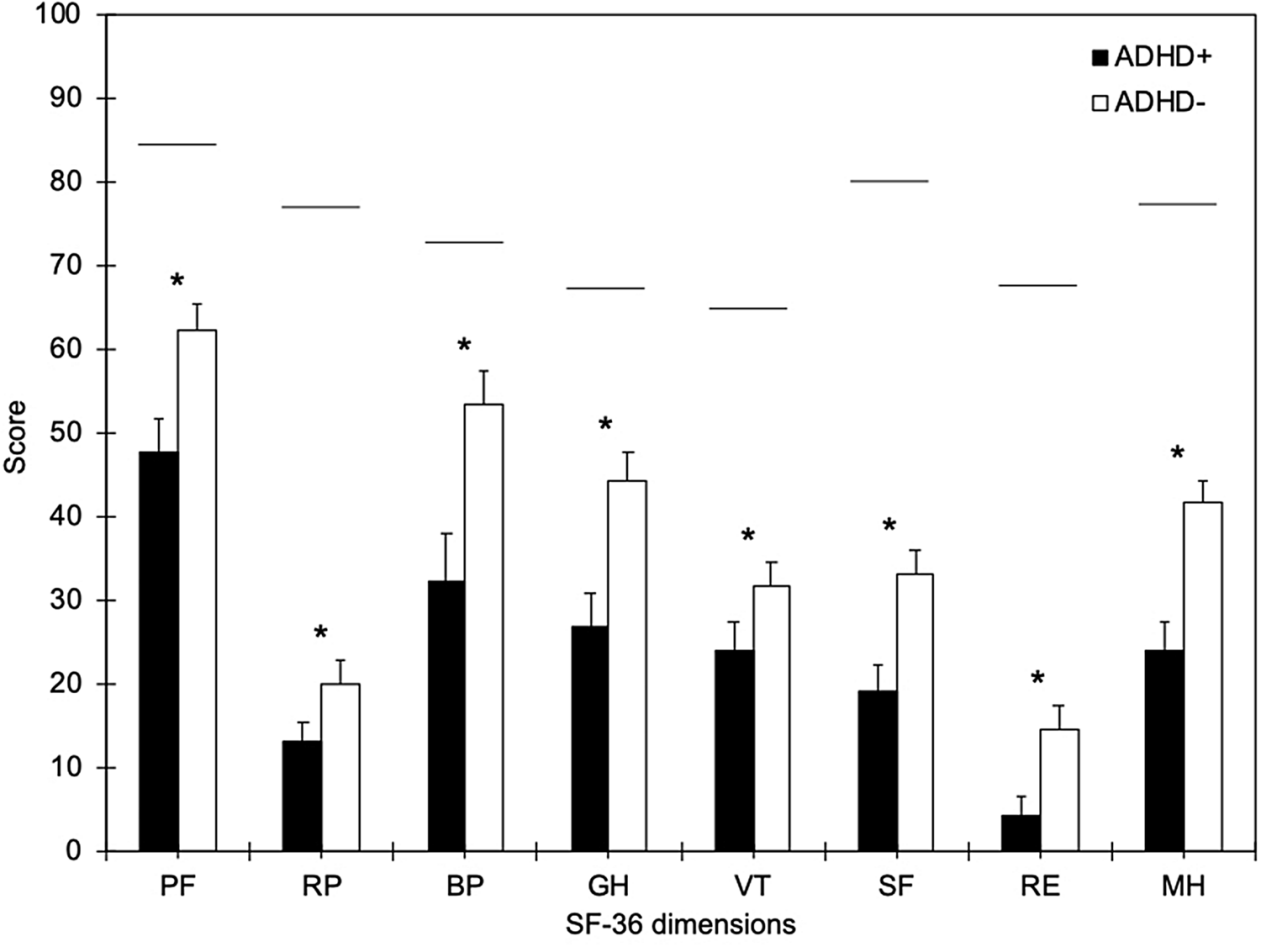
SF-36 scores in patients positive (ADHD+ , black boxes) and negative (ADHD−, white boxes) to the ASRS v1.1 Symptom Checklist Part A. The SF-36 scores ranged from 0 (worst score) to 100 (best score). All the dimensions were significantly worse in ADHD+ than ADHD– patients (* marks *p* < 0.05). Horizontal bars indicate the median score in the reference Italian population (Apolone and Mosconi 1988). Vertical error bars equal 1 S.E.M. *ADHD* attention-deficit/hyperactivity disorder, *BP* bodily pain, *GH* general health, *MH* mental health, *PF* physical functioning, *RE* role emotional, *RP* role physical, *SF* social functioning, *SF-36* Short Form-36, *VT* vitality

All SF-36 dimensions had lower score than the reference Italian population (Apolone and Mosconi 1988; Fig. 3). SF-36 scores were significantly lower in the ADHD + group than the ADHD− one for all SF-36 dimensions (PF: *p* = 0.018; RP: *p* = 0.021; BP: *p* = 0.015; GH: *p* = 0.009; VT: *p* = 0.027; SF: *p* = 0.005; RE: *p* = 0.011; MH: *p* = 0.006; Fig. 3).

GHQ-12 score was not significantly different between the two groups (ADHD + : 7.9 ± 3.7, median 8.5; ADHD−: 7.0 ± 3.5, median 7.5; *p* = 0.23), with GHQ-12 score ≥ 4 in 24 ADHD + (80.0%) and 35 (76.1%) ADHD− patients (*p* = 0.69).

### Relationship between neuropsychological and QoL measures

Among SF-36 dimensions, only GH (Spearman’s *ρ* correlation coefficient = 0.40, *p* = 0.012) and MH (Spearman’s *ρ* correlation coefficient = 0.42, *p* = 0.009) were significantly correlated with the executive function *Z*-score (i.e., the worse the cognitive measure, the lower the QoL score), while the other cognitive domains did not influence SF-36 scores.

Neuropsychological *Z*-scores did not differ in patients with GHQ-12 score ≥ 4 (verbal memory: − 0.20 ± 0.75; visuospatial memory: − 1.26 ± 1.07; working memory: − 1.24 ± 0.90; attention: − 0.50 ± 1.36; executive function: − 0.75 ± 1.69) than in those with GHQ-12 score < 4 (verbal memory: − 0.47 ± 0,73, *p* = 0.20; visuospatial memory: − 1.36 ± 0.87, *p* = 0.69; working memory: − 1.01 ± 0.96, *p* = 0.38; attention: − 0.53 ± 1.22, *p* = 0.94; executive function: − 1.20 ± 1.60, *p* = 0.32).

### Multivariate analysis

Adult ADHD, neuropsychological *Z*-scores, and variables (i.e., sex, age, education, BZD/Z-drug active principle, and DDDE) that we found to significantly influence QoL in high-dose BZD/Z-drug users in previous studies (Tamburin et al. 2017b; Lugoboni et al. 2020b) were entered as covariates in the multivariate analysis with QoL measures as dependent variables.

Positivity to adult ADHD negatively influenced (i.e., lower scores indicating worse QoL in ADHD + group) VT, SF, RE and MH; neuropsychological *Z*-scores significantly influenced (i.e., lower scores in patients with more severe neuropsychological measures) PF (working memory), RP (visuospatial memory), GH (executive function), SF (verbal and working memory, executive function), RE (visuospatial memory) and MH (executive function); age significantly influenced (i.e., higher scores indicating better QoL in older patients) all SF-36 dimensions except RE; DDDE negatively influenced (i.e., lower scores in patients using higher BZD/Z-drug dosage) PF (Table 2).

**Table 2:** Linear regression model analysis for the SF-36 domains

SF-36 domains and significant covariates	*β*	95% CI	*p* value
Physical functioning (PF), adjusted *R*^2^ = 0.85			
Working memory (*Z*-score)	6.63	0.79; 12.46	0.023
Age (years)	0.92	0.70; 1.13	< 0.001
DDDE (mg)	− 0.18	− 0.33; − 0.03	0.021
Role physical (RP), adjusted *R*^2^ = 0.71			
Visuospatial memory (*Z*-score)	5.21	0.96; 9.46	0.017
Age (years)	0.22	0.07; 0.37	0.007
Bodily pain (BP), adjusted *R*^2^ = 0.77			
Age (years)	1.01	0.88; 1.13	< 0.001
General health (GH), adjusted *R*^2^ = 0.81			
Executive function (*Z*-score)	3.97	0.16; 8.45	0.032
Age (years)	0.90	0.71; 1.09	< 0.001
Vitality (VT), adjusted *R*^2^ = 0.83			
Adult ADHD	− 6.54	− 9.90; − 4.32	0.013
Age (years)	0.53	0.42; 0.63	< 0.001
Social functioning (SF), adjusted *R*^2^ = 0.82			
Adult ADHD	− 9.35	− 18.27; − 3.54	0.037
Verbal memory (*Z*-score)	17.31	7.22; 27.38	0.001
Working memory (*Z*-score)	7.75	0.22; 15.32	0.042
Executive function (*Z*-score)	4.58	1.67; 7.48	0.003
Age (years)	0.52	0.29; 0.75	< 0.001
Role emotional (RE), adjusted *R*^2^ = 0.79			
Adult ADHD	− 5.55	− 12.57; − 1.45	0.021
Visuospatial memory (*Z*-score)	3.71	0.89; 6.52	0.011
Mental health (MH), adjusted *R*^2^ = 0.87			
Adult ADHD	− 7.11	− 20.15; − 3.24	0.008
Executive function (*Z*-score)	3.77	0.52; 7.04	0.025
Age (years)	0.86	0.71; 1.01	< 0.001

Here are reported only covariates that turned out to be significant in the multivariate linear regression analysis. Higher SF-36 scores indicate higher QoL, Adult ADHD: 0 = ADHD−, 1 = ADHD+

*DDDE* daily diazepam dose equivalent, *SF-36* Short Form-36

The multivariate logistic regression model showed that verbal (OR = 0.37, 95% CI 0.15–0.87, *p* = 0.023) and working memory *Z*-scores (OR = 0.33, 95% CI 0.19–0.57, *p* < 0.001) significantly influenced (i.e., the worse the *Z*-score, the higher the likelihood) the risk of GHQ-12 score ≥ 4, while the remaining covariates were not significant.

## Discussion

The present study confirmed that (a) screening for adult ADHD was frequently positive, (b) QoL (SF-36) was worse than the general population (Tamburin et al. 2017b), and (c) worse in ADHD + than ADHD− patients (Lugoboni et al. 2020b) in high-dose BZD/Z-drug users. This report yielded these new findings: (d) executive function was significantly worse in ADHD + than ADHD− patients, (e) some SF-36 dimensions were negatively influenced by executive function *Z*-score, and (f) multivariate analysis showed a complex interplay of adult ADHD and cognitive dysfunction in negatively influencing QoL measures (SF-36 and GHQ-12).

### Adult ADHD

Adult ADHD symptoms were overrepresented in high-dose BZD/Z-drug users, in that 39.4% of our sample was positive to adult ADHD screening with the ASRS v1.1 Symptoms Checklist Part A. This finding is in keeping with our previous report (Lugoboni et al. 2020b), and in accordance with studies in other SUD patients (van de Glind et al. 2013; Katzman et al. 2017), where the prevalence of adult ADHD symptoms was reported up to 8 times higher than the general population (Volkow and Swanson, 2013). At variance with the general population (Chung et al. 2019), demographic variables did not differ between ADHD + and ADHD− groups, probably because of the limited age range of our sample.

### Neuropsychological measures

Overall, BZD/Z-drug users showed negative *Z*-scores (i.e., worse cognitive performance than the general population) for all the cognitive domains.

Among neuropsychological measures, only executive function *Z*-score turned out to be significantly worse in ADHD + than ADHD− group. ADHD + group showed significantly worse performances in TMT-B, a cognitive test assessing divided attention and set-shifting. This finding is in keeping with a number of previous neuropsychological studies. Distinct profiles of attentional functioning were reported, with weak differences between ADHD subgroups, indicating gross disturbances of various attention functions in adult ADHD (Tucha et al. 2008). A selective impairment of attentional set shifting was documented in adults with ADHD (Luna-Rodriguez et al. 2018). Adult ADHD patients were found to display deficits in set shifting with medium-to-large effect size difference vs. controls (Rohlf et al. 2012). A study exploring executive function subdomains and several other neuropsychological functions to control for nonexecutive test demands showed selective problems in inhibition and set shifting, but not in other executive functions subdomains in adults with ADHD (Boonstra et al. 2010).

Our findings are also in accordance with a report on subjective cognitive complaints of adults with ADHD, who perceived attention and executive function among the most severely impacted neuropsychological domains (Fuermaier et al. 2015).

The present data appear to be in contrast with previous studies suggesting that neuropsychological difficulties in adult ADHD may not be confined to executive function and attention (Boonstra et al. 2005), but also involve memory and perceptual reasoning (LeRoy et al. 2019). Indeed, a review of meta-analyses on cognition in ADHD suggest substantial deficits across a variety of neurocognitive domains, and a moderator effect of age, with larger difference in comparison to controls in children and adults than adolescents (Pievsky and McGrath, 2018). This discrepancy might be ascribed to the long-term use of BZDs and Z-drugs, which are associated with impairment of a range of neuropsychological functions (Federico et al. 2017; Crowe and Stranks, 2018), in keeping with the extensive allosteric modulator effect of GABA-A receptor alpha subunits involved in cognition (Tan et al. 2011). We speculate that the high-dose BZD/Z-drug use might have caused a consistent reduction of the performance in some cognitive domains and lead to a floor effect on *Z*-scores, not allowing the demonstration of a concomitant detrimental effect of adult ADHD.

### The influence of adult ADHD and cognition on QoL

In keeping with previous studies, all SF-36 dimensions were worse in high-dose BZD/Z-drug users than the general population (Tamburin et al. 2017b), and worse in ADHD+ than ADHD− groups (Lugoboni et al. 2020b). GHQ-12 scores did not differ according to the positivity to adult ADHD screening that is in contrast to a previous report from our group (Tamburin et al. 2017b). The main reason for the discrepancy between the two studies is the stricter inclusion criteria (i.e., no major psychiatric disorders, alcohol use or other SUD) to avoid a bias effect on cognitive outcomes in the present report. Adult ADHD patients and high-dose BZD/Z-drug users frequently have multiple psychiatric comorbidities (Katzman et al. 2017), which may have a higher effect on GHQ-12 than SF-36, because the latter provides a very limited coverage of themes identified by people with mental health problems (Brazier et al. 2014).

Lower executive function *Z*-scores resulted in worse GH and MH dimensions of SF-36 in our sample. Since this is the first study to explore the correlation between cognition and QoL in high-dose BZD/Z-drug users, our finding is difficult to compare to previous reports, being the effect on SF-36 scores also related to the underlying disease and the age class. Indeed, similar findings were reported in a sample of patients with mild traumatic brain injury with a similar age than ours (Yousefzadeh-Chabok et al. 2019).

The multivariate model showed that adult ADHD and cognitive dysfunction negatively influenced QoL measures with a complex interplay between these factors and age. Positivity to adult ADHD screening negatively influenced VT, SF, RE and MH SF-36 components, in keeping with previous evidence that adult ADHD symptoms are differentially related to specific aspects of QoL (Gjervan et al. 2014). Cognitive function impairment had a differential negative influence on some SF-36 dimensions, i.e., verbal memory influenced SF, visuospatial memory influenced RP and RE, working memory influenced PF and SF, and executive function influenced GH, SF and MH. Age had a positive effect (i.e., better QoL in older patients) on all SF-36 dimensions except RE and DDDE had a detrimental effect on PF only. These findings appear to be novel in the context of cognitive deficits in adult ADHD, because previous studies mainly focused on the effect of executive dysfunction on SF-36 outcomes but did not explore the full range of neuropsychological domains (Stern et al. 2017; Sjöwall and Thorell 2019).

The multivariate logistic model indicated that lower verbal and working memory *Z*-scores were associated to increased risk of more severe psychological distress (i.e., GHQ-12 score ≥ 4), in keeping with evidence that higher GHQ-12 score is associated with cognitive deficits (Bauermeister and Bunce, 2015).

### Limitations

The main limitation is that adult ADHD was detected with the ASRS v1.1 Symptoms Checklist Part A, which is one of the recommended screening tools for adult ADHD in SUD (Crunelle et al. 2018) because of the good accuracy and short application time that might be important in the clinical setting (Dakwar et al. 2012; van de Glind et al. 2013), but does not replace a more extensive diagnostic examination. Another limitation is that adult ADHD was not subtyped (i.e., predominantly inattentive, predominantly hyperactive-impulsive, combined), but cognitive dysfunctions were not reported to be consistently different across these subtypes (Tucha et al. 2008; LeRoy et al. 2019). Moreover, our comprehensive battery of objective neuropsychological tests (Tucha et al. 2015) included the main domains, but did not assess some ADHD−related behavioral features (e.g., reward responsivity, emotional dysregulation, and temporal discounting) and basic processes that have been hypothesized to contribute to higher order cognitive dysfunction in adult ADHD (Butzbach et al. 2019). Future studies should include a larger number of neuropsychological measures (Fuermaier et al. 2019). Furthermore, the absence of a control group from the general population and a group of ADHD patients without concurrent use of BZD/Z-drugs may limit the interpretation of the present findings, in that we explored relative differences between BZD/Z-drug high-dose users with vs. without ADHD. Finally, BZD/Z-drug dosage and long-term use duration had no effect on neuropsychological measures, suggesting that a population with larger variability (i.e., low and high dose) of BZD/Z-drugs users would offer further support to these findings.

## Conclusion

We provided new information on the interplay between adult ADHD, cognitive dysfunction and QoL in in high-dose BZD/Z-drug users. These findings underscore the importance of assessing adult ADHD, neuropsychological measures and QoL in this SUD to offer a full scenario of these patients, who are frequently impaired in everyday activities (Fuermaier et al. 2017a). Moreover, they suggest that these outcomes should also be explored in other populations of long-term BZD/Z-drug users with neurological and psychiatric comorbidities. Future studies should explore whether specific cognitive rehabilitation programs alongside the standard SUD treatments (i.e., psychotherapy, pharmacotherapy, and multimodal treatment) may be effective on the neuropsychological impairment and QoL in ADHD + high-dose BZD/Z-drugs users. Whether pharmacological treatment for ADHD with methylphenidate (Mattos et al. 2013; Fuermaier et al. 2017b) and BZD/Z-drug detoxification (Soyka 2017; Tamburin et al. 2017a) might improve cognitive dysfunction and QoL in these patients is another topic for future research.

## Data Availability

A full dataset of data and statistical code is available from the corresponding author at reasonable request contingent on approval from the Ethics Committee of the Provinces of Verona and Rovigo based at Verona University Hospital.
